# Continual Learning Objective for Analyzing Complex Knowledge Representations

**DOI:** 10.3390/s22041667

**Published:** 2022-02-21

**Authors:** Asad Mansoor Khan, Taimur Hassan, Muhammad Usman Akram, Norah Saleh Alghamdi, Naoufel Werghi

**Affiliations:** 1 Department of Computer and Software Engineering, National University of Sciences and Technology, Islamabad 44000, Pakistan; asad.mansoor@ceme.nust.edu.pk (A.M.K.); taimur.hassan@ku.ac.ae (T.H.); usman.akram@ceme.nust.edu.pk (M.U.A.); 2 Center for Cyber-Physical Systems (C2PS), Department of Electrical Engineering and Computer Science, Khalifa University, Abu Dhabi 127788, United Arab Emirates; naoufel.werghi@ku.ac.ae; 3 Department of Computer Sciences, College of Computer and Information Sciences, Princess Nourah Bint Abdulrahman University, P.O. Box 84428, Riyadh 11671, Saudi Arabia

**Keywords:** continual learning, complex knowledge representations, catastrophic forgetting, multimodal datasets

## Abstract

Human beings tend to incrementally learn from the rapidly changing environment without comprising or forgetting the already learned representations. Although deep learning also has the potential to mimic such human behaviors to some extent, it suffers from catastrophic forgetting due to which its performance on already learned tasks drastically decreases while learning about newer knowledge. Many researchers have proposed promising solutions to eliminate such catastrophic forgetting during the knowledge distillation process. However, to our best knowledge, there is no literature available to date that exploits the complex relationships between these solutions and utilizes them for the effective learning that spans over multiple datasets and even multiple domains. In this paper, we propose a continual learning objective that encompasses mutual distillation loss to understand such complex relationships and allows deep learning models to effectively retain the prior knowledge while adapting to the new classes, new datasets, and even new applications. The proposed objective was rigorously tested on nine publicly available, multi-vendor, and multimodal datasets that span over three applications, and it achieved the top-1 accuracy of 0.9863% and an F1-score of 0.9930.

## 1. Introduction

Deep learning is evolving rapidly, and it has not only revolutionized computer vision, but many fields of engineering, medicine [1], and business analytics [2]. However, there still is a long way to go in order to effectively deploy deep networks in the real world to assist humans in their daily workloads in diverse aspects. Humans have the capability to adapt to any environment and excel in it where such evolution is not limited or defined by the finite dataset or pre-defined tasks for any particular domain (application). Rather, it is a lifelong learning process that evolves over time. This poses major limitations to the performance of deep neural networks.

To overcome such limitations, researchers have proposed different knowledge distillation strategies [3], end-to-end learning [4], learning without forgetting [5], expert gating [6], indefinitely long-term learning (iCaRL) [7], and hierarchical incremental learning [8]. More recently, Tian et al. [9] analyzed the shortcomings of the Kullback–Leibler (KL) divergence in learning structured representative knowledge that exhibits complex dimensional interdependencies for which the KL divergence turned out to be undefined. Furthermore, they proposed a contrastive representative objective that captures the higher correlation and output dependencies of representational data for different knowledge transfer tasks.

Mirzadeh et al. [10] proposed a multi-stage knowledge distillation strategy involving intermediate models to bridge the gap between teacher–student networks by showing that a student of a fixed size with an arbitrarily large teacher network results in a significant reduction in performance. The researchers emphasized the fact that such a topology is beneficial for mitigating the loss of information during the drastic model compression that happens when such a model is employed in resource-constrained edge devices and embedded sensor nodes. Reference [11] proposed a similar knowledge distillation approach where the backbone and the final classification layers were treated separately with the primary focus on the backbone knowledge transfer to the student. This allowed for the termination of the loss weight as the task head of the student can be trained independently afterwards. Son et al. [12] exploited multi-stage knowledge distillation by using multiple teacher and teacher’s assistant networks at each stage and introduced stochastic teaching for the student network by randomly dropping one of the many teacher and teacher assistant networks, thus regularizing the learning of the student network in the process.

An information-theoretic framework encompassing variational information maximization for knowledge transfer between a teacher and a student was presented in [13] that maximizes the mutual information between both networks to alleviate the problems associated with the knowledge distillation and transfer learning tasks. This technique bypasses the need for manual matching of the network’s activations. However, large accurate teacher networks do not necessarily generate accurate student networks as the growth capacity of the teacher is often absent in the student. This results in a failure by the student to emulate the teacher network’s performance, resulting in the increase of the training loss [14]. In such scenarios, early stopping of the teacher network can be an effective solution in contrast to multi-stage knowledge distillation steps that allow the student to converge its training loss [14]. Yaun et al. [15] exploited the fact that a teacher network with significantly less performance than the student can still effectively improve the student network due to the inherent property of knowledge distillation acting as a type of label-smoothing regularization. Thus, they proposed a teacher-free knowledge distillation strategy that allows the student network to learn from itself in the absence of any strong teacher networks while still achieving comparable results without any computational overhead.

Lee et al. [16] presented a scheme to overcome catastrophic forgetting by leveraging large, unlabeled, and easily accessible data through confidence-based sampling by which the new model instance learns the prior and current task by distilling knowledge from three sources: its previously trained instance, the newly trained teacher, as well as their ensemble. Reference [17] proposed a novel technique to combat the effects of catastrophic forgetting by first training a separate model for the new classes and then combining the old and the newly trained model using a novel double-distillation approach that relies on publicly available unlabeled data, thus mitigating the effects of the original data being unavailable. GEM (2017) [18] is another replay-based method that is composed of a memory component that stores a few dataset samples from the previous tasks, and it optimizes them by determining the direction of the change of weights. However, the optimization comes at the cost of excessive computational power and time (because the number of tasks increases over time). To mitigate these shortcomings, A-GEM (2019) was proposed in [19].

Srinidhi Hegde et al. [20] introduced a new loss term for knowledge distillation as the variational loss function, which optimizes based on four different parameters: likelihood, hint from the teacher, variational term, and block sparse regularization for transferring sparsity from the teacher to student network. It uses Bayesian neural networks, based on the principle of the determination of random variables of the parameters, which are used with the weight terms in the propagation. Contrary to the existing literature, we found three main aspects that have not been thoroughly addressed before in the context of continual learning.

The first one is the scarcity of incremental learning systems for multimodal analysis. There are frameworks that incrementally learn the data representations from similar datasets. However, the true objective of developing an incremental learning system is to identify similar data representations even from entirely different types of input, which is a hallmark of human learning. For example, a doctor can confirm a diagnosis from different data across different modalities. In fact, such an approach increases the reliability of the diagnosis. However, one hurdle in such a scheme for multimodal analysis is that such an analysis cannot be performed at the same time as the data from the different modalities become gradually available over time.

The second aspect that is addressed in this paper is the development of a system that effectively learns about unrelated tasks across different applications. During transfer learning, we fine-tuned the model that had already been trained on another dataset for our desired application. However, the fine-tuned model may no longer perform well on the previous task even if the last classification layer remains unchanged. This problem arises due to the limitation of deep learning models to catastrophically forget the old knowledge. In contrast, we humans can adapt to any environment without losing prior knowledge. Solutions have been developed that have yielded promising results to overcome catastrophic forgetting while incrementally learning the newer, related tasks [9,16,18,19]. However, to the best of our knowledge, there is no literature available to date in which the effects of catastrophic forgetting are minimized for entirely different tasks belonging to entirely different applications, for example diagnosing a disease, detecting a contraband item, detecting a cat in a picture, etc.

The third area that is explored in this paper is the analysis of the transferability of incremental learning systems on related inter- and intra-modality tasks. To summarize, the major highlights of the paper are as follows.

## 2. Contribution

This paper presents a novel approach to exploit the incremental learning tendency of deep neural networks on continuously varying environments by learning the complex relationships between different knowledge representations. Such cross-dataset and cross-domain learning is achieved by minimizing the proposed continual learning loss that encompasses the mutual distillation objective to resolve the complex knowledge dependencies. The proposed objective function was rigorously tested on nine different publicly available datasets spread across three completely independent domains, where it achieved an accuracy of 0.9863 and an F1-score of 0.9930. Furthermore, it caused a 90.63% reduction in the memory requirements over the traditional transfer learning approach without compromising the classification performance. To further highlight the generalization capacity of the proposed objective function, this paper presents extensive experiments on intra-domain transferability, showcasing the top-1 accuracy rating of 0.9270 achieved by the ResNet101 model making use of the proposed continual learning loss.

### 2.1. Proposed Applications

Although our proposed framework is equally applicable to any application and dataset that have been considered by researchers in the past, in order to effectively demonstrate the applicability of the proposed framework, we chose three distinct, scarce, and totally independent applications in this paper related to aviation security and medical sciences. The brief background description of each application is as follows.

#### 2.1.1. Application I (Learning Classification of Contraband Items from X-ray Baggage Scans)

For the last two decades, detecting contraband items in luggage has become the prime concern of aviation authorities all over the world. In order to screen the baggage against suspicious items, X-ray imagery is the tool of choice. However, due to the texture-less nature of X-ray scans, it is extremely difficult to identify these contraband items even for a human expert when they are concealed, occluded, and closely cluttered [21]. Many researchers have developed decision support systems to identify contraband items from X-ray baggage scans [22,23], and a detailed review on this can be seen in [24]. In this paper, we incrementally modeled the deep network to identify suspicious items periodically from multi-vendor X-ray scans. Afterwards, we present the classification performance of the proposed framework (trained incrementally) for this application separately, as well as on the joint application modeling. It should be noted that while this particular application primarily involves an object detection framework, we treated it as an image classification problem so that it is aligned with other applications.

#### 2.1.2. Application II (Learn to Predict Pneumonia from Chest X-ray Scans)

Pneumonia is an acute respiratory disorder that causes inflammation in the lung alveoli, resulting in painful breathing while also limiting the oxygen intake [25]. According to World Health Organization (WHO), pneumonia is the second greatest infection that causes a high number of deaths in children under the age of five throughout the world. In 2017, pneumonia was responsible for 808,694 child deaths, which accounts for 15% of all (under five years) child morality worldwide (WHO). Pneumonia can be effectively predicted through chest X-ray scans, as it is one of the most widely used modalities for diagnosing pneumonia along with listening to the respiratory patterns through a stethoscope. Many people have presented computer-aided diagnostic solutions to predict pneumonia from chest X-ray scans [26]. Similarly, in this paper, we molded our incremental learning framework to predict pneumonia-affected scans from chest X-rays.

#### 2.1.3. Application III (Classification of Retinal Diseases from Multimodal Imagery)

The third application that we considered for the evaluation of the proposed model is the classification of retinal diseases or retinopathy from retinal fundus and OCT imagery. Retinopathy tends to damage the retina, which is the innermost layer of the eye responsible for producing vision, which results in severe visual impairments including blindness. The most commonly occurring retinal diseases are diabetic macular edema (DME) and age-related macular degeneration (AMD). DME is caused due to hyperglycemia in diabetic subjects, where blood vessels become thin and start leaking fluid or protein deposits within the macula of the retina. This fluid accumulation causes retinal thickening and the formation of hard exudates, which leads towards non-recoverable loss of vision [1]. AMD, as the name suggests, is a retinal syndrome that mostly occurs in elderly people as a result of aging. Although AMD alone cannot cause blindness, it can severely degrade vision. AMD is caused due to the formation of drusen within the retina. With the progression of the disease, atrophic profiles are observed (a condition termed geographic atrophy) and blood vessels from choroids intercepting the retina, causing choroidal neovascularization. Both retinal fundus and OCT imagery are used for the evaluation of DME and AMD pathologies due to their non-invasive nature [27]. However, OCT is clinical preferred over fundus imagery because of its ability to give early and objective visualization of retinal pathologies. Nevertheless, the findings from fundus images for the severity analysis of retinal pathology cannot be fully ignored [1]. Many researchers have developed retinal computer-aided diagnostic solutions for the mass screening of retinopathy cases [26,28]. More recently, researchers have utilized retinal lesions for lesion-aware diagnosis and grading of retinopathy [1,29]. In this paper, we also modeled our framework to classify retinopathy subjects by incrementally training it on both retinal fundus and OCT scans. In addition, we applied our framework to test its classification performance on all three applications. More details about the different experimentations and their results are presented in Section 5 (Results Section).

**Figure 1:**
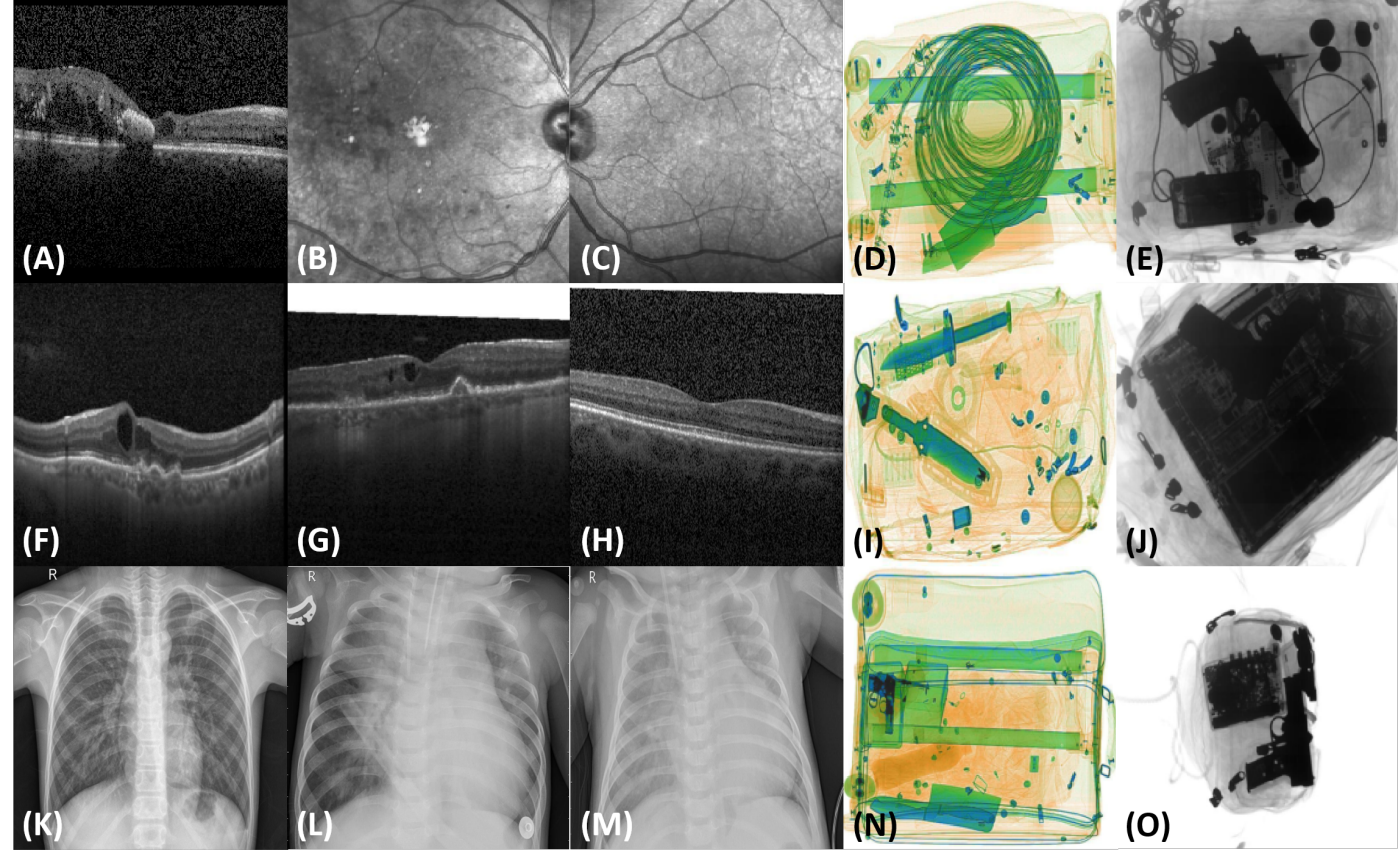
Example images showcasing different classification tasks. (**A**,**F**–**H**) show retinal OCT images of DME, wet AMD, dry AMD, and a healthy subject, respectively. (**B**,**C**) show retinal fundus scans of DME and a healthy subject, respectively. (**K**–**M**) show chest X-ray scans of pneumonic patents, and the rest of the scans showcase the examples of suspicious items in colored (**D**,**I**,**N**) and grayscale (**E**,**J**,**O**) X-ray scans.

Figure 1 depict different scans from all these applications showcasing different classification tasks that deep learning (driven by the proposed loss function) can perform in one go.

## 3. Proposed Framework

**Figure 2:**
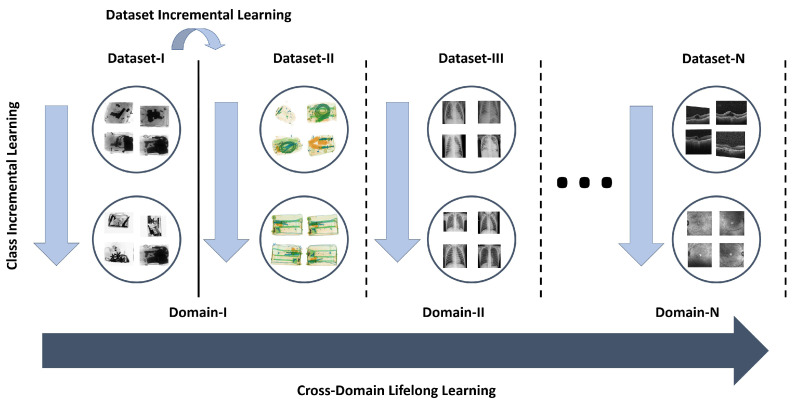
Block diagram of the proposed framework. The proposed framework can mainly be split into two phases where, in the first phase, it performs class incremental learning for a specific problem/dataset, while in the second phase, it learns about new tasks from a multimodal dataset spanning different applications without forgetting the prior knowledge.

The block diagram of the proposed framework is shown in Figure 2. Inspired by iCaRL [7], we also present a decision support system that learns indefinitely from representational data over time. However, compared to [7], the proposed framework is not only limited to class incremental learning. Instead, it can learn about new tasks from a multimodal dataset spanning different applications without forgetting the prior knowledge. This is achieved by jointly optimizing two objectives collectively termed the mutual distillation loss. The first objective allows the framework to remember prior knowledge, while the second objective allows the framework to learn about new tasks with the assumption that in real life, all the tasks have an interdependence on each other irrespective of their nature. We tried to exploit that mutual information in order to make the deep neural network learn more as humans do. The mutual distillation loss was further utilized along with the independent representation of older and newer knowledge in a continual learning objective. The detailed formulation of mutual distillation and continual learning loss is presented below.

### 3.1. Mutual Distillation Loss

Mutual distillation loss ($L_{MD}$) is a combination of two objectives that jointly optimize the deep network to effectively learn new information from a small dataset without forgetting prior learned knowledge. Contrary to all the knowledge distillation frameworks in the literature that handle catastrophic forgetting by optimizing prior and new knowledge simultaneously, but independently of each other, we assumed that real-life events have complex relationships and dependencies on each other. We tried to exploit and maximize that mutual information for effective learning.

For any deep network having an input *x* where $x\in {ℜ}^{2}$ (the superset of all samples from all applications), $L_{MD}$ is computed by jointly optimizing two objectives, which are derived from the joint distributions p(old,new)=p(old|new)×p(new) and p(new,old)=p(new|old)×p(old) where $old=f_{old}\left(x\right)$ (old knowledge soft representation) and $new=f_{new}\left(x\right)$ (soft representation of newer knowledge).

These distributions ensure that the network learns and understands the complex relationship between older and newly acquired knowledge and can effectively perform well on each task without losing its past knowledge or capping its capacity to learn new experiences. Adding the notion of classes in the above definitions of the joint distribution yields:

(1) $$p\left(old,new|t\right)=\sum_{i=0}^{w-1}p\left(old|new,t=t_{i}\right)p\left(new|t=t_{i}\right)$$

(2) $$p\left(new,old|t\right)=\sum_{i=0}^{w-1}p\left(new|old,t=t_{i}\right)p\left(old|t=t_{i}\right)$$

where *w* represents the total number of classes. Afterwards, the posterior for each class $t_{i}$ is computed through Bayes’ rule:

(3) $$p\left(t=t_{i}|old,new\right)=\frac{p\left(old,new|t=t_{i}\right)p\left(t=t_{i}\right)}{\sum_{i=0}^{w-1}p\left(old,new|t=t_{i}\right)}$$

and:

(4) $$p\left(t=t_{i}|new,old\right)=\frac{p\left(new,old|t=t_{i}\right)p\left(t=t_{i}\right)}{\sum_{i=0}^{w-1}p\left(new,old|t=t_{i}\right)}$$

where $n_{t_{i}}$ represents the number of events having outcome $t_{i}$, $s_{n}$ denotes the total events, and the prior is computed as: $p\left(t=t_{i}\right)=\frac{n_{t_{i}}}{s_{n}}$. Moreover, the likelihood of the representational data is modeled through a multivariate Gaussian distribution:

(5) $$N\left(\hat{y}|\mu ,\Sigma \right)=\frac{1}{\sqrt{2\pi^{d}\left|\Sigma \right|}}\exp-\frac{1}{2}{\left(\hat{y}-\mu \right)}^{T}\Sigma^{-1}\left(\hat{y}-\mu \right)$$

(6) $$\mu =\frac{1}{s_{n}}\sum_{i=0}^{s_{n}-1}\hat{y};\Sigma =\frac{1}{s_{n}-1}\sum_{i=0}^{s_{n}-1}\left(\hat{y}-\mu \right){\left(\hat{y}-\mu \right)}^{T}$$

where *y*∈ [$f_{old}\left(x\right),f_{new}\left(x\right)$], *d* denotes the multivariate dimension, and μ and Σ represent the mean and covariance of the distribution, respectively. Taking the log on both sides yields:

(7) $$\log p\left(t=t_{i}|old,new\right)=\log\frac{p\left(old,new|t=t_{i}\right)\times p\left(t=t_{i}\right)}{p(old,new|t)}$$

Afterwards, $L_{MD}$ for outcome $t_{i}$ is defined by minimizing $\log p(t=t_{i}|new,old)$ and $\log p(t=t_{i}|old,new)$:

(8) $$\begin{array}{c} L_{MD}\left(t_{i}\right)=-\sum_{j=0}^{n_{1}-1}f_{old}^{True}\left(x_{j}\right)/\tau \log p\left(t=t_{i}|old,new\right)/\tau \\ -\sum_{j=0}^{n_{2}-1}f_{new}^{True}\left(x_{j}\right)/\tau \log p\left(t=t_{i}|new,old\right)/\tau \end{array}$$

where τ denotes the temperature and $n_{1}$ and $n_{2}$ denote the number of old and new training samples, respectively. We can see here that Equation (8) can also be interpreted as a combination of the categorical cross-entropy loss.

### 3.2. Continual Learning Loss

$L_{MD}$ bridges the gap between new and prior knowledge by exploiting their representational dependencies. Furthermore, to make the network aware of their exclusive characteristics, we jointly optimized two more objectives such that:

(9) $$L_{CL}=L_{N}+L_{MD}+L_{O}$$

where:

(10) $$L_{O}=-\frac{1}{b_{s}}\sum_{i=0}^{b_{s}}\sum_{j=0}^{w-1}f_{new}^{True}\left(x_{i,j}\right)\log\left(p\left(old|t=t_{i,j}\right)\right)$$

and:

(11) $$\begin{array}{c} L_{N}=\frac{1}{b_{s}}\sum_{i=0}^{b_{s}}\sum_{j=0}^{w-1}f_{new}^{True}\left(x_{i,j}\right)\log\left(\frac{f_{new}^{True}\left(x_{i,j}\right)}{p(new|t=t_{i,j})}\right) \end{array}$$

where $b_{s}$ denotes the batch size. From Equations (10) and (11), we can see that $L_{O}$ is simply a categorical cross-entropy loss and $L_{N}$ is a KL divergence loss.

## 4. Experimental Setup

The experimental details of our proposed framework are as follows.

### 4.1. Datasets

The unique element of the proposed framework is that it involves different unrelated applications for which a decision support system is trained to mimic human behaviors and their learning patterns. For each application, the proposed framework was incrementally trained on different datasets containing multimodal imagery. Here, we explain all the datasets for which the proposed framework was trained.

#### 4.1.1. Application I: Classifying Baggage X-ray Scans as Having Contraband Items

Application I in the proposed study is related to the classification of baggage X-ray scans as having contraband items. The two main datasets for this problem were GDXray [30] and SIXray [23], which are explained in the following:

The *GDXray* dataset is primarily designed for non-destructive testing, and it contains 19,407 X-ray scans for the welds, casting, baggage, nature, and settings categories. However, we only chose the baggage category in the proposed study as this is only relevant for this application. The baggage group comprises 8150 grayscale X-ray scans containing guns, shurikens, knives, and razors. This dataset has been thoroughly annotated by the experts as well.

The *SIXray* dataset is one of the largest security X-ray imagery datasets for suspicious baggage item detection. The dataset contains 1,059,231 colored X-ray scans from which 8929 scans are positive (containing one or more suspicious items such as guns, knives, scissors, pliers, wrenches, and hammers). Moreover, to handle the class imbalance situation, the dataset can be arranged into three subsets, i.e., SIXray10, SIXray100, and SIXray1000 [23]. SIXray10 contains 8929 positive scans and 10× negative scans; SIXray100 contains all 8929 positive scans and 100× negative scans; SIXray1000 contains 1000 positive scans and all the negative scans [23]. Moreover, the dataset also contains detailed bounding box annotations for the suspicious items.

Please note that these datasets were originally prepared to test the frameworks for detecting contraband items from the baggage X-ray scans. However, in the proposed study, we classified the scans within these datasets as having contraband items (such as guns, knives, shuriken, pliers, scissors, hammers, wrenches, or razors) or not. The reason for doing this is because in this study, we only used a single classification model that is able to learn different tasks from different applications.

#### 4.1.2. Application II: Classification of Pneumonia from Chest X-ray Scans

In order to classify pneumonia, we used the publicly available Zhang dataset [26], which contains 1349 training scans from healthy subjects and 3883 training scans from pneumonia patients. Furthermore, the dataset contains 234 healthy and 390 pneumonia-affected scans for testing purposes. All the scans were marked against the pathologies in the respective folders.

#### 4.1.3. Application III: Classification of Retinal Diseases from Retinal Fundus and OCT Imagery

The third application on which the proposed framework was evaluated is the classification of retinal diseases from retinal fundus and OCT imagery. For this application, we used six publicly available datasets, which are briefly discussed below:

The *Rabbani* dataset [31] is one of the unique retinal image databases that contains a total of 4241 OCT and 148 fundus scans from 50 normal-, 48 dry AMD-, and 50 DME-affected subjects. The dataset was acquired at Noor Eye Hospital, Tehran, Iran. It is one of the few datasets that contains both fundus and OCT scans for each subject to correlate disease-specific features from different modalities.

The *BIOMISA* dataset [32] is another multimodal dataset to analyze retinal diseases. The dataset was introduced by the Biomedical Image and Signal Analysis Lab at the National University of Sciences and Technology, Islamabad, Pakistan. The BIOMISA dataset contains a total of 5324 OCT (657 dry AMD, 2195 ME, 904 normal, 407 wet AMD, and 1161 CSR) and 115 fundus scans from 99 subjects (8 dry AMD, 19 wet AMD, 31 ME, 24 CSR, and 17 healthy). The scans within the BIOMISA dataset were acquired through the Topcon 3D OCT 2000 at the Armed Forces Institute of Ophthalmology, Rawalpindi Pakistan.

The *Zhang* OCT dataset [26] is one of the largest retinal OCT datasets containing 109,309 scans representing wet AMD (choroidal neovascularization), dry AMD (drusen), DME, and healthy subjects. The dataset was arranged in a way that 108,309 scans were used for training and 1000 scans for testing. The scans in the Zhang dataset were acquired by Spectralis, Heidelberg Inc.

The *Duke-I* dataset [33] is one of the largest datasets from the Vision and Image Processing (VIP) lab, Duke University, USA. It contains 38,400 retinal OCT scans from healthy and AMD subjects. The scans were acquired by Spectralis, Heidelberg Inc. The dataset also contains annotations for aiding the automated tools in extracting retinal layers.

The *Duke-II* dataset [34] contains 610 OCT scans from severe DME subjects. The dataset was first introduced in [34] by the VIP lab, and it contains detailed markings for the retinal layers and fluids from two expert clinicians. The scans within Duke-II were acquired by Spectralis, Heidelberg Inc.

*Duke-III* [35] is the third dataset from the VIP lab that we used in this research. The dataset contains retinal OCT scans from 15 AMD subjects, 15 DME subjects, and 15 healthy subjects. The dataset was primarily designed for the classification of these pathologies, and the scans within the datasets are organized with respect to the pathologies that they reflect. The scans within the Duke-III dataset were also acquired by Spectralis, Heidelberg Inc.

### 4.2. Evaluation Metrics

Standard classification metrics such as accuracy, sensitivity, specificity, and precision were utilized to measure the performance of the proposed framework. Moreover, to produce unbiased results during the imbalanced situation (especially with the SIXray dataset [23]), we also used the F1-score.

### 4.3. Training and Implementation Details

The classification models used in the proposed study were developed using Keras APIs on the Anaconda platform. The proposed framework runs incrementally where, in each iteration, the classifier learns incrementally about new tasks within each application. The training process in each iteration was conducted for 20 epochs. The optimizer used during the training was ADADELTA. The proposed framework was developed and tested on a Lambda Labs Tensorbook with the following specifications: Core i7-9750H@2.6 GHz, 32 GB DDR4 RAM, and NVIDIA RTX 2080 Max-Q GPU. At each iteration, the model continually learns from the previously trained instance using its logits (which were converted to soft probabilities). The source code of the proposed framework will be released publicly at the following link: https://github.com/anonymous/anonymous (accessed on 27 December 2021). Moreover, the training–testing dataset split in the proposed study was 1 to 4, i.e., 20% of the scans were used for training and 80% for testing purposes.

## 5. Results

We evaluated the proposed framework for three different applications involving nine publicly available datasets. While ResNet101 provided the best results for all the different datasets, the proposed framework was also evaluated with MobileNet [36], ResNet50 [37], and VGG-16 [38]. In addition, the fine-tuning comparisons were performed with ResNet101, as it was the best-performing model in the group.

We started with training the classification model on the GDXray dataset in which we incrementally added each suspicious class with the small training dataset. We continued the same process for the SIXray dataset and then for different applications. Our motivation here was to make the classification model effectively learn the complex relationships between different tasks during the knowledge distillation process. For that, we used our proposed continual learning loss, which explicitly uses the mutual distillation objective to resolve the dependencies between the prior knowledge and the new information. Furthermore, as other knowledge distillation frameworks, $L_{CL}$ also retains the past knowledge by minimizing $L_{O}$ and effectively learns new tasks through $L_{N}$. We also compared $L_{CL}$ with other popular loss functions such as the KL divergence and multi-category cross-entropy to see how effectively it learns the newer knowledge while retaining the already learned task. The evaluations on each dataset individually and then on the combined dataset are presented below.

### 5.1. Evaluations on the GDXray Dataset

The baggage scans in the GDXray dataset contain all the suspicious items, so for the first training iteration, the model was just trained for predicting whether a grayscale scan contains a gun or not. Afterwards, in each successive iteration, contraband items such as knives, shurikens, and razors were added.

**Figure 3:**
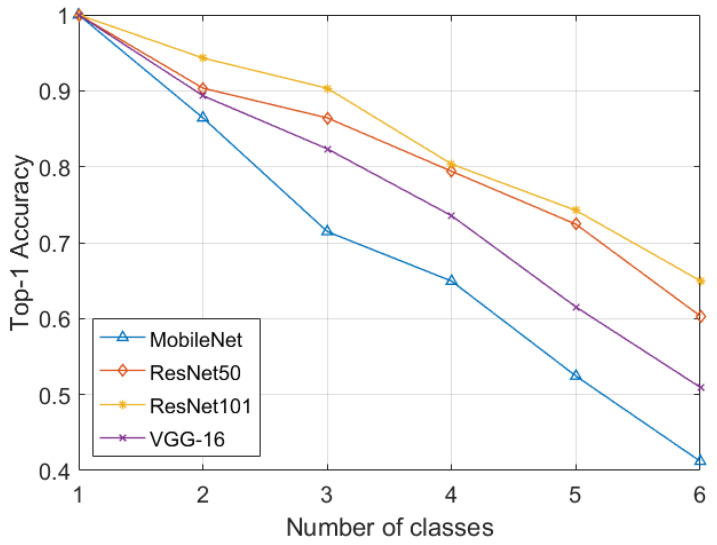
Incremental classification performance in terms of the top-1 accuracy on GDXray.

Furthermore, to correctly classify scans containing miscellaneous and normal items such as springs, paperclips, etc., within the GDXray dataset, we added a separate normal class. In some images, the suspicious items may occur in groups. Therefore, a “multiple” class was added for such cases, bringing the total number of classes to six. The performance on GDXray in each iteration was measured through accuracy, sensitivity, specificity, precision, and F1-scores. From Table 1, it can be observed that the proposed framework had an accuracy of 0.6498% and an F1-score of 0.7462 for the six6-class suspicious item classification when employing ResNet101. we can observe that the proposed framework accurately recognized the suspicious item scans with an accuracy of 0.6498% and an F1-score of 0.7462 using ResNet101. Moreover, we also computed the classification results through ResNet101-based transfer learning on GDXray and compared its classification performance with the proposed incremental learning strategy. In order to fine-tune ResNet101 on the GDXray dataset, we used the cross-entropy loss function as the proposed objective function was designed for continual learning schemes. It can be observed from Table 1 that the proposed framework lagged only 5.72% from the transfer learning approach in terms of the F1-score. Moreover, the performance of class incremental learning can be observed in Figure 3, where we can observe how effectively the proposed objective function retained the prior knowledge and produced classification results extremely comparable to the fine-tuning approach.

### 5.2. Evaluations on the SIXray Dataset

After training and evaluation on GDXray, we incrementally trained the proposed framework on SIXray to identify suspicious items from colored X-ray scans.

**Figure 4:**
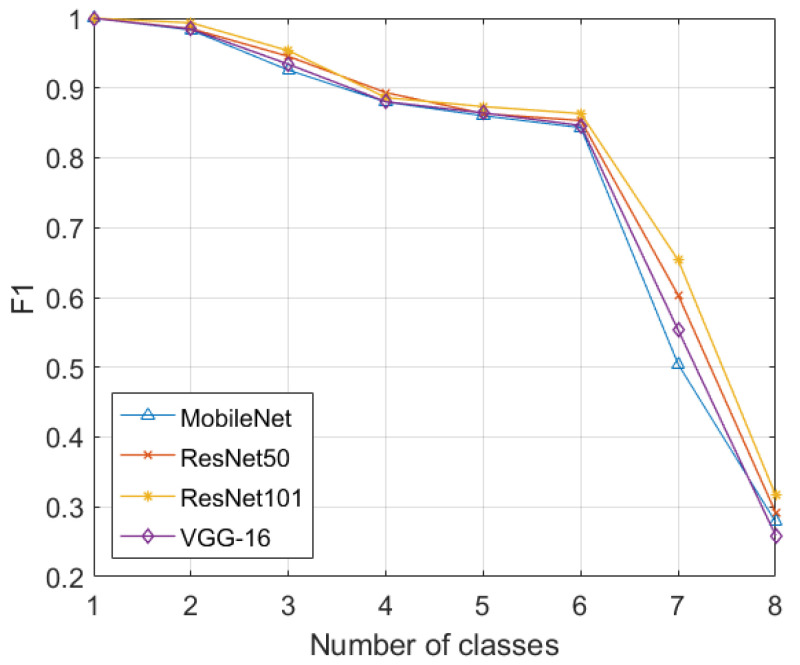
Incremental classification performance in terms of the F1-score on SIXray.

At first, the deep learning models were trained to recognize the scans containing guns. In the next iterations, the models were incrementally trained to recognize the knives, wrenches, pliers, scissors, and hammers. In a similar manner to the GDXray dataset, for the scans containing multiple suspicious items, we added a separate class for them, namely “multiple”. In the last iteration, the deep learning models were trained to identify negative (normal) scans that did not contain any suspicious items. It should be noted here that the SIXray dataset is highly imbalanced (i.e., it contains 8929 suspicious scans and 1,050,302 normal scans). Moreover, Table 2 shows the classification performance of the proposed framework on the SIXray dataset, where it can be observed that the proposed framework achieved an accuracy of 0.9663 and an F1-score of 0.3167. The significant difference in these scores is due to the fact that SIXray dataset is extremely imbalanced. The imbalanced nature of the SIXray dataset can be further seen in Figure 4, where the classification performance of the deep model (in terms of the F1-score) drastically decreased while learning the negative samples (in the eighth iteration). Apart from this, we can also observe the sudden decrease in the classification performance in the seventh iteration. This is because the number of scans containing individual items is extremely low as compared to those scans that contain multiple suspicious items (i.e., there are around 8905 out of 8929 positive scans within the SIXray dataset that contain multiple suspicious items), thus creating a class imbalance situation, yielding the significant decrease in the classification performance. However, even the fine-tuning approach suffered from such an imbalance, as we can observe the 17.77% decrease from the sixth to seventh iteration in Figure 4.

Furthermore, we evaluated all the deep learning models on SIXray10, SIXray100, and SIXray1000 using the proposed objective function, and it lagged by 32.14% on SIXray10, 49.47% on SIXray100, and 50.93% on SIXray1000 from the transfer learning approach in terms of the F1-scores, as evident from Table 3. These results again should be analyzed with the fact that the SIXray dataset is highly imbalanced.

### 5.3. Evaluations on the Zhang Chest X-ray Dataset

For the Zhang chest X-ray dataset, we trained the deep learning models on healthy chest X-ray scans first and then incrementally trained them to identify the pneumonia-affected scans. We also computed the results through the transfer learning approach on the Zhang chest X-ray dataset using ResNet101 and multi-category cross-entropy. From Table 4, we can see that the proposed framework lagged from the transfer learning approach only by 2.26% in terms of the accuracy while using a significantly smaller training dataset at one time in memory.

### 5.4. Evaluations on the Rabbani Dataset

For the third application, we trained the deep learning models first on the Rabbani dataset. It is one of the few datasets that allows retinal examination and screening of retinal subjects based on multimodal imagery. First of all, the proposed framework was trained on healthy OCT and fundus scans. In the subsequent iterations, it was trained on OCT and fundus scans of the DME and AMD pathologies, respectively. The classification performance of the proposed framework on the Rabbani dataset is shown in Table 5, where we can see that the proposed objective had a gap of only 4.65% (in terms of accuracy) to bridge the gap between the incremental learning and transfer learning approach.

### 5.5. Evaluations on the BIOMISA Dataset

BIOMISA is one of the recently published datasets containing retinal fundus and OCT images of healthy and retinopathy-affected subjects. The scans within the BIOMISA dataset were acquired by the Topcon 3D OCT 2000 machine. The performance of the proposed framework on the BIOMISA dataset is shown in Table 6, where it can be observed that the best performance was achieved for the ResNet101 model where it only lagged from fine-tuning by 1.008% in terms of the F1-score.

### 5.6. Evaluations on the Duke Datasets

To evaluate the proposed framework on the Duke datasets, we first merged Duke-I and Duke-II together because Duke-I only contains AMD and normal pathologies, whereas Duke-II only contains DME pathologies. Therefore, training the system solely on Duke-II for classifying only the DME subjects would not have been appropriate. Apart from this, we evaluated Duke-III separately since it contains scans from all three pathologies. The classification performance of the proposed framework on the Duke datasets is shown in Table 7 and Table 8. From Table 7 and Table 8, we can observe that the proposed framework was able to achieve an F1-score of 0.9537 on the Duke-I and Duke-II datasets and an F1-score of 0.9584 on the Duke-III dataset using the ResNet101 model.

### 5.7. Evaluations on the Zhang OCT Dataset

The last dataset on which the proposed framework was evaluated is the Zhang OCT dataset. In total, 1000 scans were used for the evaluation purposes (250 scans per pathology) as per the dataset standard, and the proposed framework was able to achieve an accuracy of 0.9260 using ResNet101, as evident from Table 9. Moreover, it can also be noted from Table 9 that the proposed framework only lagged from the fine-tuning approach by 3.44%, which is less significant especially considering the fact that the proposed framework drastically reduces the memory and data requirements for training as compared to the fine-tuning approach.

### 5.8. Evaluations on Combined Dataset

**Figure 5:**
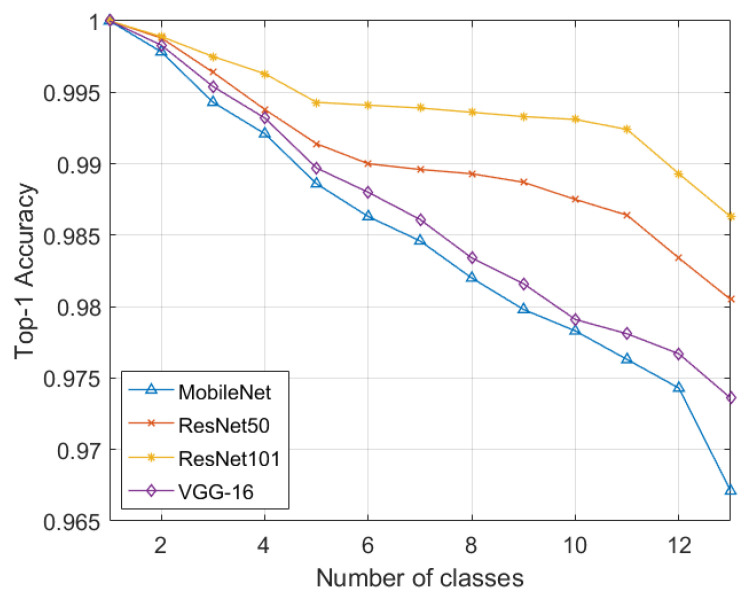
Incremental classification performance in terms of the top-1 accuracy on the combined dataset.

Apart from evaluating the proposed objective function individually on each dataset, we trained the deep learning models on the combined dataset with 13 classes, namely guns, knives, shurikens, razors, pliers, scissors, hammers, wrenches, normal, multiple items, pneumonia, AMD, and DME, where the proposed objective function was utilized in making the models effectively learn new classes for different tasks across different domains. Table 10 shows the classification performance of the deep learning models encompassing the proposed objective function for effectively learning different classification tasks. It can be observed that with the ResNet101 model, the proposed objective was able to achieve the top-1 test accuracy of 0.9863, which lagged by the transfer learning approach with only a 0.0045% difference in terms of accuracy. It should be further noted that while achieving a comparable classification performance, the incremental learning scheme (powered by the proposed objective function) resulted in a 90.63% decrease in the dataset at one time in memory for training as compared to the transfer learning approach. Moreover, Figure 5 further shows the incremental training performance of the deep learning models. We also compared the performance of the proposed loss function with the multi-category cross-entropy loss and KL divergence loss, as shown in Table 11. All these loss functions were utilized during the incremental training, where the proposed continual incremental loss was able to produce 15.88% better results in terms of accuracy on the combined dataset as compared to the categorical cross entropy.

**Figure 6:**
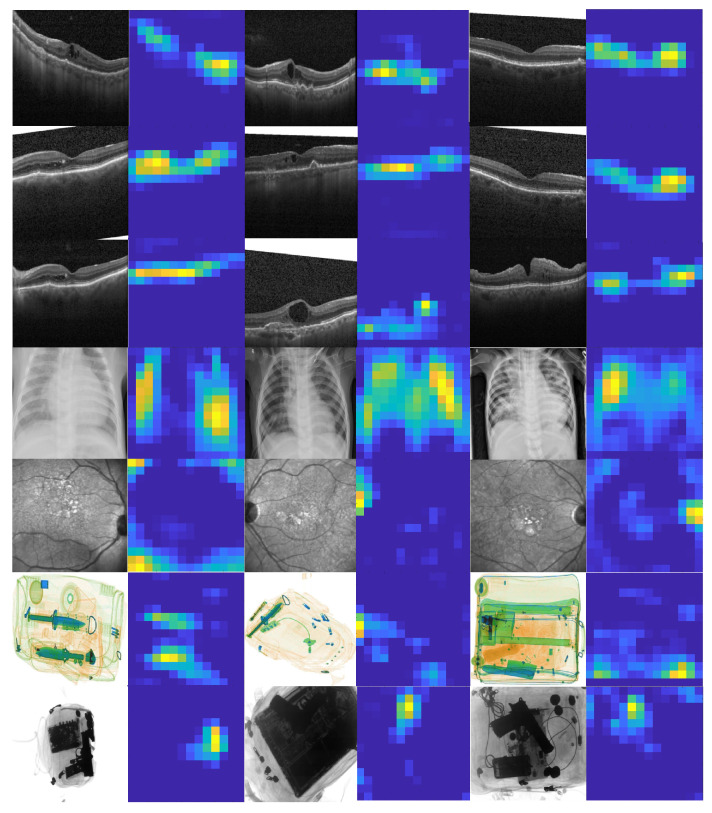
Learned representations on the combined dataset.

Apart from this, we also performed an intra-domain transferability analysis to further examine the generalization capacity of the deep learning models trained using our proposed objective function. It can be observed from Table 12 that the incrementally trained models achieved good transferability performance for the retinal image datasets. However, for the SIXray and GDXray datasets, the performance was on the lower bounds. This is because of the significant variability in the image features (as, for instance, GDXray X-ray scans are grayscale in nature, whereas SIXray scans are colored). We did not include the Zhang chest X-ray dataset in this experiment since it was a single dataset for the pneumonia classification domain in the proposed study. Moreover, we did not evaluate inter-domain transferability since such experiment would be less valuable (e.g., it would not be meaningful if the model trained to identify suspicious baggage items predicts retinal diseases). In addition to this, we display the final probability maps of the random scans (from the nine publicly available datasets) in Figure 6 to analyze how effectively the ResNet101 model (trained using the proposed objective function) interpreted them. It can be observed from Figure 6 how robustly the ResNet101 model retained the important representations for the correct classifications irrespective of the scan type, scan modality, or scan acquisition machinery. Furthermore, it can be analyzed how well the learning was achieved over the totally unrelated tasks across different domains.

### 5.9. Ablation Study

The proposed study involved two ablative aspects, which are as follows.

#### 5.9.1. Impact of Mutual Distillation Loss

The backbone of the proposed $L_{CL}$ is the mutual distillation loss $L_{MD}$, which is minimized during the training of the deep learning. Mutual distillation loss is crucial for learning complex knowledge representations, which avoids catastrophic forgetting by understanding the complex relationships between old and new knowledge. We trained the ResNet101 model incrementally, and during the training, we excluded the mutual distillation loss to see its adverse effects. It can be observed from Table 11 that on each dataset, there was a significant performance degradation if the complex dependencies between old and new knowledge (especially on the same dataset) were not analyzed.

#### 5.9.2. Dependency on Temperature

**Figure 7:**
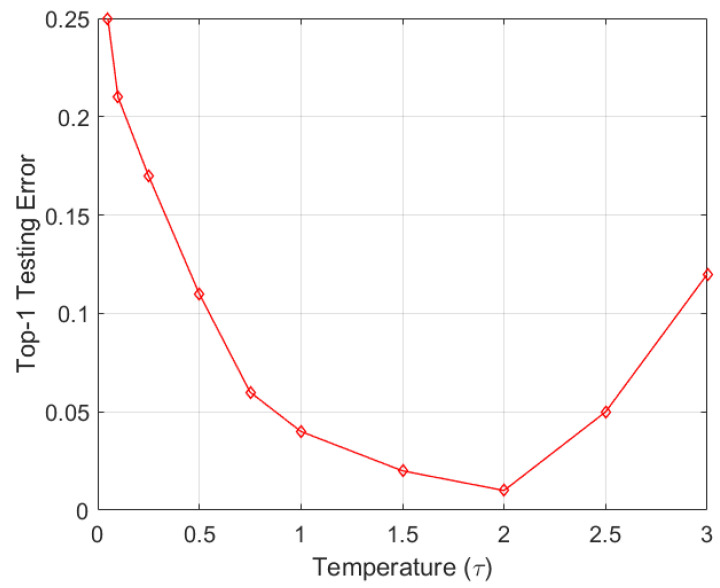
Effect of τ in terms of the top-1 testing error.

In order to have a trained model that retains its ability to keep its prior knowledge, soft probabilities are useful. They also greatly help in diversifying deep learning models to learn new representations. The second ablative aspect of the proposed study was to vary the temperature to see its effects on the overall learning. The temperature τ was used to scale the values of a layer before the Softmax activation was applied to obtain probabilistic outputs from the model. Figure 7 shows the experiment results for the ResNet101 model on the combined dataset, where we can observe that the best learning was achieved for τ=2. It can be seen that scaling of the values should not be too lenient or too aggressive, which results in performance degradation. The temperature is, of course, dataset dependent and should be computed separately for each dataset empirically, as the values may differ. Here, we can analyze that a temperature ranging from 1.5–2 is ideal for effective learning on the combined dataset.

## 6. Conclusions

This paper presented the continual incremental objective that incorporates mutual distillation loss to effectively solve the complex representational dependencies between prior and new knowledge, which significantly reduces the chances of catastrophic forgetting in deep neural networks. The proposed objective was evaluated on different pre-trained models and on nine publicly available datasets across three different applications/domains involving different learning tasks. The proposed framework was able to achieve comparable performance as transfer learning, reducing by 90.63% the memory requirements during model training. The proposed objective function is currently susceptible to class imbalance situations (as observed in the evaluations on the SIXray dataset), and in the future, it can be extended to weight the imbalanced dataset samples for more effective and robust training.

## Figures and Tables

**Table 1:** Performance comparison on GDXray. The proposed framework with ResNet101 [37] achieved comparable performance to the fine-tuned model.

Metric	M [36]	R50 [37]	R101 [37]	VGG-16 [38]	FT
Acc	0.4121	0.6033	0.6498	0.5095	0.7147
TPR	0.5166	0.7282	0.7709	0.6632	0.8109
TNR	0.2019	0.3520	0.4062	0.2004	0.5211
PPV	0.5656	0.6933	0.7231	0.6252	0.7730
F1	0.5400	0.7103	0.7462	0.6436	0.7915

Acc: accuracy, TPR: sensitivity, TNR: specificity, PPV: precision, M: MobileNet, R50: ResNet50, R101: ResNet101, and FT: fine-tuning using ResNet101 driven by the cross-entropy loss.

**Table 2:** Performance comparison on SIXray. The drastic decrease in the F1-score as compared to the accuracy is a reflection of the imbalance of the dataset.

Metric	M [36]	R50 [37]	R101 [37]	VGG-16 [38]	FT
Acc	0.9607	0.9626	0.9663	0.9573	0.9903
TPR	0.8973	0.9122	0.9229	0.8827	0.9608
TNR	0.9612	0.9630	0.9667	0.9579	0.9906
PPV	0.1647	0.1738	0.1912	0.1518	0.4651
F1	0.2783	0.2919	0.3167	0.2590	0.6268

Acc: accuracy, TPR: sensitivity, TNR: specificity, PPV: precision, M: MobileNet, R50: ResNet50, R101: ResNet101, and FT: fine-tuning using ResNet101 driven by the cross-entropy loss.

**Table 3:** Performance comparison on $L_{CL}$ on the SIXray subsets in terms of the F1-score. All scores were computed for the best-performing ResNet101 model.

Subset	Proposed	FT
SIXray10	0.5042	0.7431
SIXray100	0.3167	0.6268
SIXray1000	0.1814	0.3697

**Table 4:** Performance comparison on the Zhang chest X-ray dataset. The proposed framework with ResNet101 [37] achieved comparable performance to the fine-tuned model.

Metric	M [36]	R50 [37]	R101 [37]	VGG-16 [38]	FT
Acc	0.6362	0.8109	0.8333	0.6619	0.8526
TPR	0.7359	0.8692	0.8769	0.7487	0.8897
TNR	0.4701	0.7137	0.7607	0.5171	0.7906
PPV	0.6983	0.8350	0.8593	0.7210	0.8763
F1	0.7166	0.8518	0.8680	0.7346	0.8830

Acc: accuracy, TPR: sensitivity, TNR: specificity, PPV: precision, M: MobileNet, R50: ResNet50, R101: ResNet101, and FT: fine-tuning using ResNet101 driven by the cross-entropy loss.

**Table 5:** Performance comparison on the Rabbani dataset. The proposed framework with ResNet101 [37] achieved comparable performance to the fine-tuned model.

Metric	M [36]	R50 [37]	R101 [37]	VGG-16 [38]	FT
Acc	0.9050	0.9104	0.9206	0.9183	0.9655
TPR	0.9030	0.8966	0.9021	0.9154	0.9680
TNR	0.9083	0.9327	0.9504	0.9231	0.9615
PPV	0.9409	0.9556	0.9671	0.9506	0.9760
F1	0.9216	0.9252	0.9335	0.9326	0.9720

Acc: accuracy, TPR: sensitivity, TNR: specificity, PPV: precision, M: MobileNet, R50: ResNet50, R101: ResNet101, and FT: fine-tuning using ResNet101 driven by the cross-entropy loss.

**Table 6:** Performance comparison on the BIOMISA dataset.

Metric	M [36]	R50 [37]	R101 [37]	VGG-16 [38]	FT
Acc	0.8734	0.9069	0.9277	0.8898	0.9421
TPR	0.8727	0.9047	0.9242	0.8881	0.9381
TNR	0.8757	0.9149	0.9405	0.8959	0.9568
PPV	0.9619	0.9745	0.9824	0.9685	0.9874
F1	0.9152	0.9383	0.9524	0.9266	0.9621

Acc: accuracy, TPR: sensitivity, TNR: specificity, PPV: precision, M: MobileNet, R50: ResNet50, R101: ResNet101, and FT: fine-tuning using ResNet101 driven by the cross-entropy loss.

**Table 7:** Performance comparison on the Duke-I and Duke-II datasets. The two datasets were combined due to the overlap of the classes.

Metric	M [36]	R50 [37]	R101 [37]	VGG-16 [38]	FT
Acc	0.8784	0.9228	0.9361	0.9016	0.9607
TPR	0.8812	0.9296	0.9360	0.9087	0.9575
TNR	0.8716	0.9067	0.9362	0.8846	0.9684
PPV	0.9421	0.9594	0.9721	0.9492	0.9863
F1	0.9107	0.9443	0.9537	0.9285	0.9717

Acc: accuracy, TPR: sensitivity, TNR: specificity, PPV: precision, M: MobileNet, R50: ResNet50, R101: ResNet101, and FT: fine-tuning using ResNet101 driven by the cross-entropy loss.

**Table 8:** Performance comparison on the Duke-III dataset. The proposed framework with ResNet101 [37] achieved comparable performance to the fine-tuned model.

Metric	M [36]	R50 [37]	R101 [37]	VGG-16 [38]	FT
Acc	0.8734	0.9069	0.9277	0.8898	0.9421
TPR	0.8727	0.9047	0.9242	0.8881	0.9381
TNR	0.8757	0.9149	0.9405	0.8959	0.9568
PPV	0.9619	0.9745	0.9824	0.9685	0.9874
F1	0.9152	0.9383	0.9524	0.9266	0.9621

Acc: accuracy, TPR: sensitivity, TNR: specificity, PPV: precision, M: MobileNet, R50: ResNet50, R101: ResNet101, and FT: fine-tuning using ResNet101 driven by the cross-entropy loss.

**Table 9:** Performance comparison on the Zhang OCT dataset. The proposed framework with ResNet101 [37] achieved comparable performance to the fine-tuned model.

Metric	M [36]	R50 [37]	R101 [37]	VGG-16 [38]	FT
Acc	0.8410	0.9060	0.9260	0.8770	0.9590
TNR	0.8080	0.8920	0.9133	0.8507	0.9560
TPR	0.9400	0.9480	0.9640	0.9560	0.9680
PPV	0.9758	0.9809	0.9870	0.9831	0.9890
F1	0.8840	0.9344	0.9488	0.9121	0.9722

Acc: accuracy, TPR: sensitivity, TNR: specificity, PPV: precision, M: MobileNet, R50: ResNet50, R101: ResNet101, and FT: fine-tuning using ResNet101 driven by the cross-entropy loss.

**Table 10:** Performance comparison on the combined dataset. Even with cross-domain classes, the proposed model achieved comparable performance to the fine-tuned model while requiring fewer resources.

Metric	M [36]	R50 [37]	R101 [37]	VGG-16 [38]	FT
Acc	0.9671	0.9805	0.9863	0.9736	0.9908
TPR	0.9687	0.9809	0.9865	0.9744	0.9910
TNR	0.8549	0.9522	0.9692	0.9188	0.9780
PPV	0.9978	0.9993	0.9995	0.9988	0.9997
F1	0.9831	0.9900	0.9930	0.9864	0.9953

Acc: accuracy, TPR: sensitivity, TNR: specificity, PPV: precision, M: MobileNet, R50: ResNet50, R101: ResNet101, and FT: fine-tuning using ResNet101 driven by the cross-entropy loss.

**Table 11:** Performance comparison of the KD divergence, cross-entropy (CE), and *L_CL_* (with and without *L_MD_*) on different datasets.

Loss	GDXray	SIXray	Chest	Rabbani	BIOMISA	Duke-I, II	Duke-III	Zhang	Combined
KL	0.5341	0.9267	0.7836	0.8671	0.8739	0.9142	0.9419	0.9190	0.8451
CE	0.5196	0.9061	0.7580	0.8422	0.8654	0.9273	0.9442	0.9110	0.8296
*L_CL_* with *L_MD_*	0.6498	0.9663	0.8333	0.9206	0.9277	0.9361	0.9532	0.9260	0.9863
*L_CL_* without *L_MD_*	0.5194	0.9145	0.7603	0.8361	0.8597	0.8759	0.8942	0.8696	0.7643

**Table 12:** Intra-domain transferability analysis. Zhang here only represents the Zhang OCT dataset.

Training → Testing	Top-1 Accuracy	Classified Samples
SIXray → GDXray	0.6537	5089/7784
GDXray → SIXray	0.5175	438,579/847,405
Duke → Zhang	0.9270	927/1000
Zhang → Duke	0.9095	30,567/33,608
Rabbani → Zhang	0.8560	856/1000
Zhang → Rabbani	0.8439	2986/3538
Rabbani → Duke	0.8241	27,697/33,608
Duke → Rabbani	0.8685	3073/3538
Rabbani → BIOMISA	0.8252	2809/3404
BIOMISA → Rabbani	0.8035	2843/3538
BIOMISA → Zhang	0.8170	817/1000
Zhang → BIOMISA	0.8172	2782/3404
BIOMISA → Duke	0.8202	27,568/33,608
Duke → BIOMISA	0.8022	2731/3404

## Data Availability

SIXray: A Large-scale Security Inspection X-ray Benchmark for Prohibited Item Discovery in Overlapping Images. https://github.com/MeioJane/SIXray. Large Dataset of Labeled Optical Coherence Tomography (OCT) and Chest X-Ray Images https://data.mendeley.com/datasets/rscbjbr9sj/3. Rabbani Dataset https://www.biosigdata.com. BIOMISA Dataset https://biomisa.org/index.php/downloads/. Duke Datasets https://people.duke.edu/~sf59/software.html.
